# Resequencing Composite Kazakh Whiteheaded Cattle: Insights into Ancestral Breed Contributions, Selection Signatures, and Candidate Genetic Variants

**DOI:** 10.3390/ani15030385

**Published:** 2025-01-29

**Authors:** Aigerim K. Khamzina, Alexander V. Igoshin, Zhadyra U. Muslimova, Asset A. Turgumbekov, Damir M. Khussainov, Nikolay S. Yudin, Yessengali S. Ussenbekov, Denis M. Larkin

**Affiliations:** 1 Green Biotechnology and Cell Engineering Laboratory, Kazakh National Agrarian Research University, Almaty 050010, Kazakhstan; aigerim.khamzina55@gmail.com; 2 Institute of Cytology and Genetics of the Siberian Branch of the Russian Academy of Sciences, Novosibirsk 630090, Russia; 3 Faculty of Veterinary and Zooengineering, Kazakh National Agrarian Research University, Almaty 050010, Kazakhstandamir.khussainov@kaznaru.edu.kz (D.M.K.);; 4 Kurchatov Genomics Center, Institute of Cytology and Genetics of the Siberian Branch of the Russian Academy of Sciences, Novosibirsk 630090, Russia; 5 Royal Veterinary College, University of London, London NW1 0TU, UK

**Keywords:** Kazakh Whiteheaded cattle, resequencing, signatures of selection, genetic variants, Kazakhstan, economically important traits

## Abstract

This study investigates the genetic architecture of the Kazakh Whiteheaded (KWH) cattle, applying population genetics approaches to resequenced genomes. *F_ST_* analysis of 66 cattle breeds identified breeds for admixture analysis. At K = 19, the composite KWH breed showed contributions from Hereford, Altai, and Kalmyk cattle. Principal component analysis and ancestry inference confirmed these patterns, with KWH genomes comprising 45% Hereford, 30% Altai, and 25% Kalmyk ancestries. Haplotype analysis revealed 73 regions under putative selection in KWH, some shared with Hereford (e.g., with the gene *DCUN1D4*) and some KWH-specific (e.g., with the gene *SCMH1*). F*_ST_* analysis identified 105 putative intervals under selection, with key genes (*KITLG, SLC9C1,* and *SCMH1*) involved in coat colour and physiological adaptations. Functional enrichment using The Database for Annotation, Visualization, and Integrated Discovery (DAVID) in selected regions highlighted clusters associated with developmental processes, ubiquitination, and fatty acid metabolism. Point F*_ST_* identified 42 missense variants in genes enriched in functions related to economically important traits. Local ancestry inference revealed genomic intervals with predominantly non-Hereford ancestry, including high Altai (e.g., *SCAPER*) and Kalmyk (e.g., *SRD5A2*) contributions, while Hereford-dominated regions included genes *ENO1* and *RERE*. This work elucidates the genomic contributions and adaptive signatures of selection shaping the KWH breed, providing candidate genetic variants for breeding program improvement and enhanced genome predictions.

## Simple Summary

The efficiency of contemporary agriculture depends on our ability to make new and modify old livestock breeds in response to climatic and demographic challenges. This can be facilitated using genetic markers that contribute to economically important traits in different environments. To start the process of finding such markers for breeds developed in the harsh climates of Kazakhstan, we sequenced the genomes of a local beef cattle breed called Kazakh Whiteheaded. We learned about their genetic ancestry from native Altai cattle, hardy Kalmyk breed, and highly productive Hereford cattle. We found genome intervals inherited from these breeds and those regions that react to the ongoing artificial selection. Importantly, we found specific mutations that can be tested for breed improvement and genomic prediction of individual animal features. Overall, this study has improved our knowledge of the genetic origins of Kazakhstan’s most popular cattle breed and provided new genomics tools for improving livestock breeding strategies in this country.

## 1. Introduction

The contemporary cattle breeds originate from at least two domestication events of Aurochs in the Middle East and Indus Valley [1]. After domestication, various locally adapted cattle populations were produced by combining farmer breeding and natural selection to local environmental conditions [2,3]. In addition, it is known that introgression with local wild bovine species has contributed to some breed formation [4], and convergent evolution events contributed to adaptation in extreme cold environments [5]. Studies of locally adapted breeds from different countries provide new information about genetic mechanisms of adaptations to different environments critical for creating the next generation of breeds, which combine the features of highly productive cosmopolitan breeds and locally adapted populations. This is critically important now when climates are changing fast. One example of ongoing climate change is the frequency of extreme conditions affecting the same territory [6]. There are locally adapted breeds that can deal with extreme cold (e.g., the Yakut cattle expressing convergent mutation in the gene *NRAP* [5]) and African native cattle breeds that developed inherent thermotolerance [7]. We must introduce these adaptations to commercial breeds suffering from climate change. This could be achieved through the formation of composite breeds or by gene editing. Either way, there is a need to know about breed adaptations’ genetic mechanisms to choose the right breed combinations and mutation donors. The genetic studies of extant composite breeds could help reveal genome intervals and traits inherited from different populations. This information could also be used to improve these breeds by applying marker-assisted or genomic selection and facilitating genomic prediction.

In the present study, we focused on the Kazakh Whiteheaded beef breed. It was developed in the USSR in 1930–1940 and has been actively used in the Republic of Kazakhstan since 1950 [8]. The breed originates from a crossing of local Kazakh cattle, native Kalmyk cattle breed, and Hereford bulls. The resulting animals acquired qualities of the ancestral populations: high adaptability, strong constitution, early maturity, and high meat yield [8]. The appearance of the Kazakh Whiteheaded cattle reminds the Hereford: coat colour is red of varying intensity, with a white head, chest, belly, lower limbs, and tail brush. There are animals with white markings on the withers and rump; the front part is better developed than the back part. There is evidence of local climate adaptation: the hair is thick and short in summer and long and slightly curly in winter [8]. Kazakh Whiteheaded cattle is the most used breed in Kazakhstan, with a total number of animals of ~500 thousand, including ~200 thousand cows (https://www.gov.kz, accessed on 27 January 2025), substantially contributing to beef production in the country [9,10].

Microsatellite and SNP array studies of Kazakh Whiteheaded cattle confirm the relationship of the breed with Hereford [11,12]. A recent genotyping study involving Kazakh Whiteheaded animals from Kazakhstan and Russia also shows links between Kalmyk cattle and local cattle from the Republic of Altai [13]. The authors hypothesised that the Altai cattle could be genetically similar to the extinct native Kazakh cattle population used for making the Kazakh Whiteheaded cattle [13]. Previous studies reported signatures of selection in the Kazakh Whiteheaded breed in the regions of the coat colour genes *KIT* and *KITLG* [14]. There have been selection signals on BTA6, in the region *LCORL-NCAPG*, associated with production traits in cattle (e.g., daily weight gain, muscle development, and carcass traits).

The purpose of this study was to go to the next step after microsatellite and SNP array studies to investigate the origins of Kazakh Whiteheaded cattle using whole-genome resequencing data to find genes under selection and, importantly, candidate genetic variants that could be tested during the efforts to improve this and other breeds. Therefore, our work focuses on genomic analysis of ~40 resequenced Kazakh Whiteheaded cattle individuals from the Republic of Kazakhstan and the Russian Federation. They were compared to world breeds from the 1000 Bull Genomes Project to confirm Kazakh Whiteheaded’s origins, their chromosomes’ local ancestry, and patterns concerning ancestral populations. We performed scans for signatures of selection and detected several novel candidate genetic variants that could be tested in marker-assisted selection of this and other breeds.

## 2. Materials and Methods

### 2.1. DNA Extraction, Genotyping, Resequencing, and Sample Selection

#### 2.1.1. DNA Extraction

Fifty-three blood samples of the Kazakh Whiteheaded (KWH) breed were collected from the Agro Baltabay farm in the Almaty region (Kazakhstan), and 25 hair follicle samples were obtained from the Elimay farm in the East Kazakhstan region. Genomic DNA extraction from blood was performed using the phenol–chloroform extraction method [15]. Blood (500 µL) was lysed with a lysis buffer and incubated at 37 °C for 10–15 min, followed by digestion with Proteinase K at 55 °C for 3 h. The mixture was extracted with phenol–chloroform, centrifuged, and the aqueous layer was used. DNA was precipitated with ethanol and sodium acetate, incubated at −20 °C for 1–2 h, washed with 70% ethanol, air-dried, and resuspended in TE buffer. For the extraction of genomic DNA from hair follicles, a commercial PureLink™ Genomic DNA Mini Kit was used according to the manufacturer’s instructions (Thermo Fisher Scientific, Waltham, MA, USA). This process involved four steps: sample preparation, DNA binding, washing, and elution of purified DNA. The average DNA yield was 1–2 µg per 1 mL of blood. Extraction using the phenol–chloroform method resulted in an average concentration of 90.4 ng/µL, while the PureLink™ extraction yielded 52 ng/µL on average, both measured using the Nanodrop 2000 spectrophotometer (Thermo Fisher Scientific, Waltham, MA, USA).

#### 2.1.2. Genotyping

DNA genotyping was performed using the BovineSNP50 v.3 array (Illumina, San Diego, CA, USA) and parsed by the GenomeStudio v.2.0.5 software (Illumina, USA; https://emea.support.illumina.com/array/array_software/genomestudio/downloads.html; accessed on 15 January 2025). The PLINK v. 1.9 program (https://www.cog-genomics.org/plink/; accessed on 15 January 2025) was used to identify genetic relatedness within genotyped individuals using the “--genome” option for all SNPs with MAF >0.01 in our dataset.

#### 2.1.3. Resequencing

We selected 35 unrelated (relatedness (PI_HAT) < 0.25; 33 dams, 2 sires) purebred individuals for sequencing using Illumina’s NovaSeq X Plus sequencing technology (paired-end 2 × 150 bp) at Novogene Co., Ltd. (Cambridge, UK) with the amount of raw sequence data >50 Gbp per sample (~15× raw coverage). In addition, 10 previously sequenced KWH samples from Altai, Russia, were obtained from the 1000 Bull Genomes Project, Run 9. All samples were mapped to the bovine reference genome (ARS-UCD1.2_Btau5.0.1Y; https://hgdownload.soe.ucsc.edu/goldenPath/bosTau9/bigZips/; accessed on 15 January 2025) following the 1000 Bull Genomes Project protocol [16]. Briefly, the reads were cleaned with Trimmomatic v.0.38 [17] in PE mode using relevant Illumina adapters and with the parameters LEADING:20 TRAILING:20 SLIDINGWINDOW:3:15 AVGQUAL:20 MINLEN:35. The cleaned reads were mapped to the reference cattle genome using BWA-MEM v.0.7.17 [18], and then duplicate reads were marked with Picard v.2.18.2 [19] with the parameter OPTICAL_DUPLICATE_PIXEL_DISTANCE = “2500” to match sequencing technology. Next, we performed a base quality score recalibration (based on the ARS1.2PlusY_BQSR_v3 dataset) and follow-up variant calling procedure using GATK v.3.8-1-0-gf15c1c3ef [20]. GVCF files of 35 KWH samples from Kazakhstan and 10 samples from Russia were merged into a VCF file using the GATK’s haplotype caller module. SNPs were filtered using “hard filtering” parameters in GATK: QD < 2.0, FS > 60.0, MQ < 40.0, MQRankSum < −12.5, and ReadPosRankSum < −8.0 [20] and LD-pruned in PLINK v.1.9 using the parameters “--indep-pairwise 100 kb 100 0.2”. The resulting 330,756 SNP positions were merged with the taurine set of breeds from the 1000 Bull Genomes Project (Run 9) to identify breeds closely related to KWH, resulting in 269,629 common SNPs.

#### 2.1.4. Selecting Samples for Comparison

**Figure 1:**
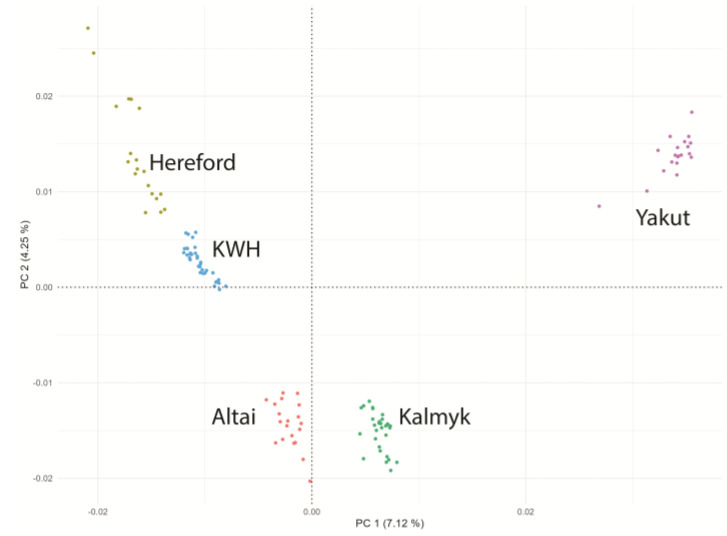
Principal component analysis of five cattle breed populations used in this study. KWH—Kazakh Whiteheaded cattle, Hereford—Hereford breed, Altai—Altai cattle, Kalmyk—Kalmyk cattle, Yakut—Yakut cattle.

We performed a Hudson F*_ST_* comparison of the KWH breed with breeds (no. samples of ≥10) in the 1000 Bull Genomes Project. Using the outcomes of this analysis, we chose 25 breeds related to the KWH based on F*_ST_* results (Table S1). For this set of breeds, ADMIXTURE v1.3.0 (https://dalexander.github.io/admixture/download.html; accessed on 15 January 2025) [21] and a set of 269,629 SNPs were used to analyse population structure and PONG [22] was used to visualise results (Figure S1). Based on ADMIXTURE and F*_ST_* results, we selected Altai, Kalmyk, and Hereford breeds as closely related to our KWH population. Yakut cattle have been selected as an outgroup. We downloaded raw sequence data for these breeds from NCBI SRA (Hereford (20) and Yakut (20)) and used our resequencing data for Kalmyk (30) and Altai (20) cattle (Table S2). These samples were aligned to the reference cattle genome, as described above, gVCF files were merged in VCF using GATK, and principal component analysis (PCA) was performed using PLINK v1.9 (https://www.cog-genomics.org/plink/; accessed on 15 January 2025) with the “--pca” option [23] to examine related populations and exclude admixed individuals. Scatter plots of closely related breeds of the first two principal components (PC1 and PC2) were generated using the ggplot2 package version 3.5.1. in R [24] to display the genetic variation and clustering patterns among the individuals in the dataset. As a result of this analysis, we excluded six KWH individuals with strong Hereford components and one Hereford individual (Table S2). Our final dataset contained 39 KWH, 19 Hereford, 30 Kalmyk, 20 Altai, and 20 Yakut samples (Figure 1).

### 2.2. F_ST_

SNPs and genome regions demonstrating divergence between the target breeds and the global cattle population may contribute to breed-specific phenotypes. To identify such SNPs and genome intervals, we used data from the taurine set of the 1000 Bull Genomes Project (1KBGP) (Run 9) to calculate F*_ST_* statistics between KWH breed, on the one hand, and related breeds, on the other. The VCFtools software (v.0.1.13; https://sourceforge.net/projects/vcftools/files/; accessed on 15 January 2025) [25] was used for the calculation with the options “*--fst-window-size 50,000 --fst-window-step 25,000 -max-missing 0.8*” for window-based analysis, and without the options “*--fst-window-size, --fst-window-step*” for individual SNPs. The SNPs found in the top 0.01% windows sorted by F*_ST_* values were considered in this analysis. For point F*_ST_* analysis, we considered SNPs with F*_ST_* values > 0.4.

### 2.3. Identification of Signatures of Selection with HapFLK Statistics and Enrichment Analysis

The hapFLK test allows the detection of selected genome intervals based on differences in haplotype frequencies between populations and considering their hierarchical structure [26]. The number of haplotype clusters (K) was estimated for the KWH breed dataset as 20, using the fastPhase software (v. 1.2) [27]. The HapFLK (v.1.4; https://forge-dga.jouy.inra.fr/projects/hapflk/files; accessed on 15 January 2025) software was used for analysis with the following parameters: “-K 20 –nfit = 30 –kfrq”. *p*-values were calculated using normal distribution as a null model with the “*MASS*” R package version 7.3-64 (“*rlm*” function) [28]. The q-values were then calculated using the “*qvalue*” R package version 2.38.0 [29]. Statistically significant intervals were defined by at least one SNP with *q*-value < 0.01, and interval boundaries were set by the first SNPs with *q*-values > 0.2 upstream and downstream of significant SNP(s). For these intervals, haplotype diversity (using the *hapflk-clusterplot. R* script) and local trees (using *local_reynolds.py* and *local_trees. R* scripts) were visualised to find breed(s) under selection.

Functional enrichment analysis for genes located in the regions of interest was performed using The Database for Annotation, Visualization, and Integrated Discovery (DAVID v.2023q4; https://davidbioinformatics.nih.gov, accessed on 15 January 2025). As suggested by the tool developers, we investigated enriched functional-related gene groups and used an enrichment score equal to 1.3 as a threshold for enriched groups (https://davidbioinformatics.nih.gov, accessed on 15 January 2025).

### 2.4. RFMix

We applied a robust forward–backwards algorithm implemented in RFMix [30] to screen for the presence of putative Hereford, Kalmyk, and Altai cattle haplotypes in autosomes of the KWH breed. This algorithm uses designated reference haplotypes to infer local ancestry in designated admixed haplotypes; thus, three breeds were selected as reference panels: Hereford, Kalmyk, and Altai. The window size was set to three (-w 3), and the option “–reanalyze-reference” with three iterations was used to analyse the reference haplotypes as if they were query haplotypes [30].

## 3. Results

### 3.1. Breed Selection for Comparative Analysis

Based on Hudson F*_ST_* analysis of 66 breeds from the 1000 Bull Genomes Project (sample size ≥10 samples + Kazakh cattle (9 samples), 26 pure breeds were selected for Admixture analysis (Table S1). At K = 3, the KWH demonstrated a clear fraction of Hereford ancestry. The origin of the other two KWH components was unclear as they were present in various breeds. However, at K = 19, KWH demonstrated the contribution of the Kalmyk cattle component and the component of Altai cattle (Figure S1). Based on these results, Hereford, Altai, Kalmyk, and Yakut (outgroup) cattle animals were selected for further analyses. Principal component analysis (PCA) was performed on animals from these breeds and the KWH samples to avoid admixed individuals. Admixed individuals were removed, resulting in a dataset containing 39 KWH, 19 Hereford, 30 Kalmyk, 20 Altai, and 20 Yakut samples (Figure 1).

### 3.2. Signatures of Selection

**Figure 2:**
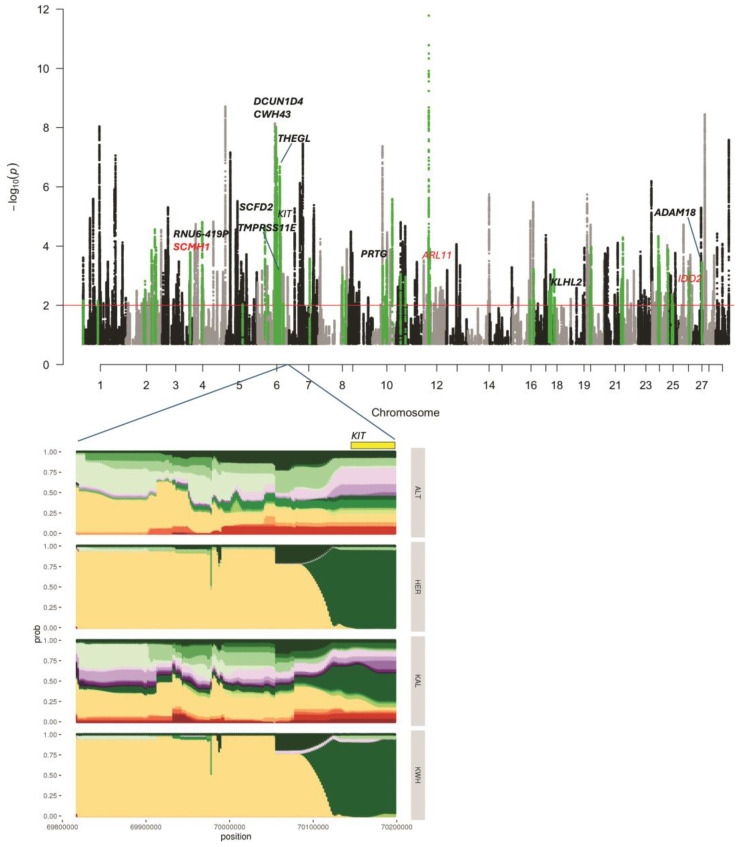
Signatures of selection identified in the four-breed set (Yakut cattle was used as an outgroup). The Manhattan plot shows the selection signatures assigned to the Kazakh Whiteheaded (KWH) lineage (in green). Genes found in putatively selected regions supported by window F*_ST_* analysis are shown in bold. In red are candidate genes with missense mutations detected in point F*_ST_* analysis with Hereford samples excluded. The significance threshold corresponds to *q*-value = 0.01. The haplotype diversity in four breeds, Altai (ALT), Hereford (HER), Kalmyk (KAL), and KWH, is shown for the putatively selected region containing the coat-colour gene *KIT*. Colours indicate different haplotypes.

The list of regions under putative selection detected in KWH by haplotype analysis (hapFLK) is shown in Table S3. This analysis revealed 73 regions (Figure 2) putatively selected in the KWH breed, of which 31 selection signals were shared with Hereford cattle. F*_ST_* analysis against all breeds resulted in 105 intervals under putative selection in the KWH. However, F*_ST_* values were relatively low (0.18–0.36) (Table S4). When one related breed was removed from the F*_ST_* analysis, we detected regions demonstrating higher allele differentiation, suggesting that the allele frequencies in the KWH could be close to the breed excluded from the F*_ST_* analysis (Tables S5–S7).

Thirteen genes were found at the intersection of haplotype and window-based F*_ST_* results (Table S3). The single-point F*_ST_* analysis revealed 42 missense variants with F*_ST_* > 0.4 when Hereford samples were excluded (Table S8). One missense variant in *SLC9C1* was detected when Kalmyk cattle were removed from the comparison (Table S9). Three missense variants from genes *ARL11, SCMH1,* and *IDO2* were located in putatively selected intervals detected by haplotype analysis, of which one (*SCMH1*) overlapped with window-based F*_ST_* analyses (Table 1 and Table S3). When comparing KWH against all breeds in F*_ST_* analysis (Table S4), the top signals were detected in the *DCUN1D4, SCMH1, RNU6-419P, ASB13*, and *KITLG* genes. The observed F*_ST_* values were low (0.23–0.27). When we excluded Hereford samples from the analysis (Table S5), *DCUN1D4* and *SCMH1* were still at the top of the list, suggesting that alleles of these genes in the KWH could be inherited from Hereford. Indeed, the haplotype analysis confirmed selection in the *DCUN1D4* region (*q*-value = 9.37 × 10^−9^), suggesting that this gene was under putative selection in the KWH and Hereford lineages. On the other hand, haplotype analysis has confirmed selection in the *SCMH1* region in the KWH lineage only (*q*-value = 0.00016). Point F*_ST_* identified a missense variant in the *SCMH1* gene Met392Val with relatively high F*_ST_* = 0.44, detected only when Hereford samples were excluded. This suggests that *SCMH1* alleles could be inherited by KWH from the Hereford and undergo further selection as indicated by haplotype analysis.

A coat-colour gene *KIT* was found in a putative haplotype signature of selection interval on BTA6 (*q*-value = 8.62 × 10^−6^), with signatures of selection in both KWH and Hereford (Figure 2). Another coat-colour gene, *KITLG*, was among the top genes in the “against-all” window F*_ST_* analysis (Table S4; F*_ST_* = 0.22), and the F*_ST_* value went up to 0.32 when Hereford samples were removed (Table S5). The top haplotype signature of selection specific to the KWH breed was observed in the region of the gene *THEGL* on BTA6. It was supported by elevated F*_ST_* values in the F*_ST_* results (Table S3). Among other genes that were located in putatively selected intervals of the KWH genome and supported by window F*_ST_* analysis were *STAP1*, *GNRHR*, *CENPC*, *RNU6-419P*, *PRTG*, *ADAM18*, *TMPRSS11E,* and *KLHL2* (Table S3). Six additional genes demonstrated signatures of selection in KWH and Hereford in the haplotype analysis and F*_ST_* results: *SCFD2*, *RASL11B*, *KCTD10*, *TEC*, *RORA,* and *SLAIN2*.

The point F*_ST_* analysis focusing on mutations with “high” or “moderate” potential effects highlighted several candidate genetic variants. One missense genetic variant with F*_ST_* = 0.43 in the gene set without Kalmyk cattle samples resulted in an Ile514Val change in the gene *SLC9C1* (Table S9). The same mutation was reported in the set lacking Hereford samples (F*_ST_* = 0.44; Table S8). There were 42 genetic variants in this set in total (Table S8). Of them, there were three variants in the genes reported in our window F*_ST_* or haplotype analyses or both: *ARL11* and *IDO2* were reported by the haplotype analysis, and the *SCMH1* was reported by haplotype and window F*_ST_* results.

Functional enrichment analysis of genes under selection exclusively in the KWH based on haplotype analysis (Table S10) highlighted one enriched cluster (ES = 1.39) with the terms “developmental protein”, “differentiation”, and “signal 5”. When we looked at the regions under putative selection in KWH and Hereford breeds (Table S11), a large, enriched cluster (ES = 1.74) included functional categories “regulation of platelet activation”, “threonine/tyrosine-protein kinase catalytic”, etc. When all haplotype-based putative selected intervals were combined (Table S12), these two clusters were present, and two additional clusters were revealed (ES = 1.40): “Ubl conjugation pathway”, “ubiquitin-dependent protein catabolic process”, “protein ubiquitination” and (ES = 1.39): “DOMAIN:SH2”, etc. Forty-two missense variants with F*_ST_* >0.4 with Hereford samples excluded from the analysis resulted in one enriched cluster (ES = 2.20) containing the terms “albumin”, “fatty acid binding”, etc. (Table S13).

### 3.3. Local Ancestry Inference (LAI)

**Figure 3:**
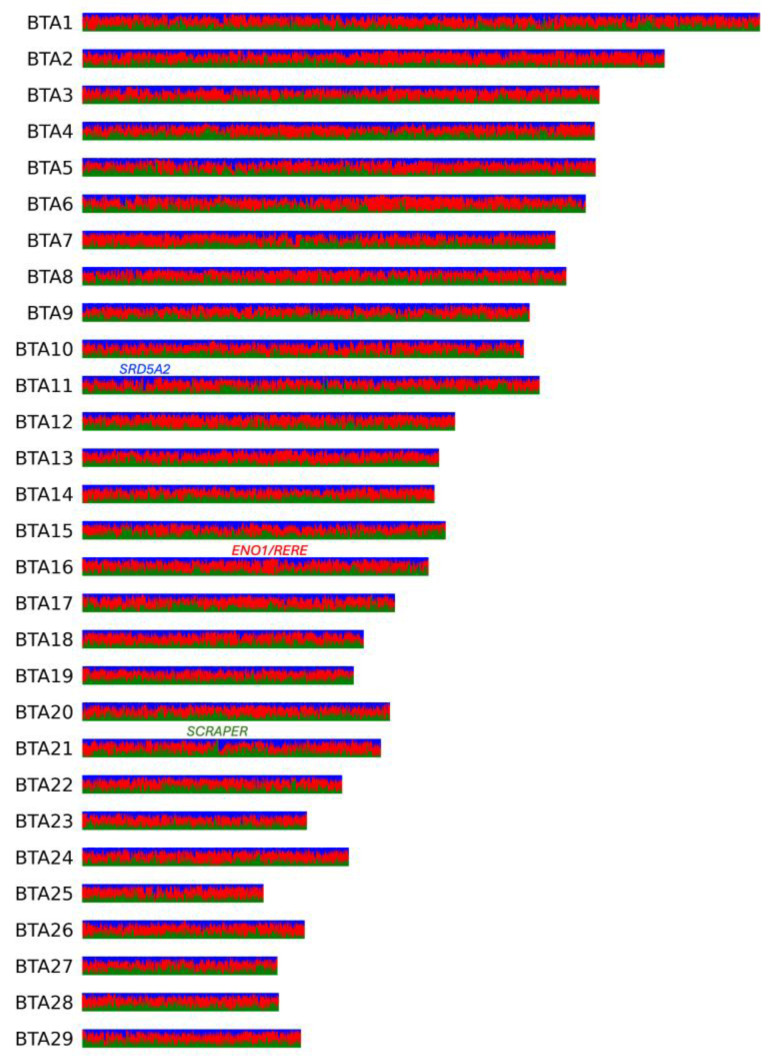
Local inference analysis for three ancestral KWH populations shown for all KWH autosomes. In red are Hereford haplotypes, in blue are haplotypes inherited from Kalmyk cattle, and in green are haplotypes inherited from native (Altai) populations. Genes found in the regions with the highest local ancestries of each of the three populations are shown.

According to LAI analysis (Figure 3, Table S14), the genome-wide average fractions of Altai (native), Kalmyk, and Hereford ancestries in the KWH genome were 30%, 25%, and 45%, respectively. The ancestry fractions in some chromosome intervals largely deviated from these values. Thus, in the genomes of KWH, 273.69 Mbp and 103.02 Mbp had >50% of native and Kalmyk ancestries, respectively. This means 376.71 Mbp of the KWH genome had <50% Hereford ancestry. In total, 26.16 Mbp of the KWH genomes had >90% non-Hereford ancestry. None of the regions were 100% inherited from native or Kalmyk cattle. Considering local estimates for Hereford ancestry, 3.05 Mbp of the genome had a fraction of >90%.

The highest fraction of Hereford ancestry (0.97) was observed on BTA16 in a region containing two annotated genes, *ENO1* and *RERE* (Figure 3). The highest fraction of native (Altai) ancestry (0.94) was observed in two intervals on BTA21, both overlapping the gene *SCAPER* (Figure 3). In the case of Kalmyk ancestry, the highest fraction (0.87) was observed on BTA11 13.8 Kbp upstream of the *SRD5A2* gene (Figure 3).

The functional enrichment in regions with >50% native (Altai) ancestry resulted in 12 clusters (ES > 1.3; Table S15). The top cluster (ES = 3.0) comprised terms related to the glycine metabolic process. Among other clusters were peptidase activity (ES = 2.16), calmodulin binding (ES = 1.97), acyl-CoA metabolic process (ES = 1.92), and defence response to bacteria (ES = 1.72). The same analysis for Kalmyk ancestry revealed six clusters (Table S16). The top one (ES = 5.43) was related to calcium ion binding. The other clusters included terms related to homeobox and embryonic skeletal morphogenesis (ES = 3.75), keratins and keratinisation (ES = 3.34), and mitosis (ES = 1.40). We then looked at the regions with >60% of Hereford ancestry and found 10 clusters (Table S17). Among them were lipid binding/transport (ES = 2.11), albumin (1.78), mitochondrion (ES = 1.40), and alkaline phosphatase (ES = 1.39).

## 4. Discussion

Our study focused on the genetics and origins of Kazakhstan’s most popular beef cattle breed, the *Kazakh Whiteheaded*. This breed was developed in the Kazakh SSR in the 1930–1940s by crossing native cattle with Hereford and Kalmyk breeds to develop productive hardy cattle. We used over forty whole-genome resequenced KWH samples to learn about this composite breed’s history, selection, and local ancestry. We hypothesised that a combination of genetics from local cattle, hardy Kalmyk, and the highly productive Hereford breed will have complementary contributions to KWH’s genetics and that contrasting the KWH genomes to their closely related or/and ancestral breeds will reveal genes and genetic variants that could be used to improve beef breeding programmes in Kazakhstan and other countries. Overall, the results confirmed that the breed has composite origins and pointed to candidate genes and genetic variants to be focused on in future efforts on breed improvement.

Comparison of the resequenced KWH samples with world breeds has confirmed a substantial contribution of Hereford genetics to KWH formation, supported by the visual similarity of the two breeds and the previous studies [13,31]. The contribution of Kalmyk and Altai cattle genetics has been confirmed but at a higher level of stratification in admixture analysis than in previous work [13]. This could be due to the small number of Kalmyk cattle individuals available in the 1000 Bull Genomes project dataset, as, in the previous studies, the close relation of these breeds and KWH was revealed at a lower level of stratification when genotyping datasets were used [13]. Interestingly, our analysis did not show close relations between the KWH and Kazakh cattle from China when using samples from the 1000 Bull Genomes dataset. A recent study from Xu et al. (2024) shows that Kazakh cattle from China have a different genetic profile from the KWH [32]. Overall, our admixture and PCA results agreed with the previous studies of KWH and provided a foundation for the follow-up analyses.

The local ancestry inference further confirmed a substantial contribution of Hereford genetics to the formation of KWH, which was higher than the contributions from Kalmyk and native (Altai) cattle. The coat-colour genes, *KIT* and *KITLG*, were found in chromosome intervals with a high fraction of Hereford ancestry, likely explaining the coat-colour similarities between the KWH and Hereford. Regions with >50% native (Altai) cattle ancestry enriched for bacterium defence pathways likely contribute to traits beneficial for survival in native environments. Kalmyk ancestry is associated with calcium ion binding, keratins and keratinisation, and embryonic skeletal morphogenesis, which are potentially linked to physical robustness, climate adaptations, and/or milk production. On the other hand, regions enriched for Hereford ancestry were associated with lipid binding, albumin, and mitochondrial functions, likely reflecting traits aligned with productivity and metabolic efficiency. Genes detected in the regions with the highest ancestry from one of the three breeds further support complementary contributions of ancestral genetics to the KWH phenotypes. For example, the *RERE/Atrophin 2* (arginine–glutamic acid dipeptide repeats) gene found in an interval with the top (0.97) Hereford ancestry was identified as a candidate affecting embryonic growth [33]. Atrophin 2 is essential for the function of various signalling centres in mouse development during embryogenesis [34]. The *SCAPER* (S phase cyclin a-associated protein in the endoplasmic reticulum), located in the top interval of Altai ancestry (0.94), is highly expressed in testis and involved in reproductive systems in both males and females [35]. It was shown that, in male mice, *SCAPER* mutants are sterile, while females have smaller litter sizes [35]. A top region with Kalmyk cattle ancestry (0.87) was found upstream of the *SRD5A2* (steroid 5 alpha-reductase 2) gene, associated with ketosis susceptibility [36] and sperm mobility, both economically important traits in livestock. Therefore, we could conclude that top genes from native populations or local breeds (Kalmyk) contributed to the reproductive efficiency of the KWH, and Hereford brought haplotypes contributing to embryonic growth.

While local ancestry inference is robust in detecting regions under purifying selection in composite breeds [37,38], this approach is not appropriate for detecting signatures of positive selection. These regions in our study were identified using single-point and haplotype approaches. As the KWH is a relatively young composite breed, there probably have not been enough generations to break ancestral haplotypes and express strong signatures of selection using a single SNP (point) approach. This could explain why our point analysis did not detect such strong signals. However, haplotype analysis identified a substantial number of signatures of selection in the KWH, including those shared between KWH and Hereford breeds, suggesting that some haplotypes could be inherited from Hereford and undergo further selection in KWH.

The top genes in selected intervals putatively inherited by KWH from Hereford were *DCUN1D4* (defective in cullin neddylation 1 domain containing 4) and *SCMH1* (scm polycomb group protein homolog 1). The *DCUN1D4* gene was previously associated with carcass traits in beef cattle breeds, including Angus [39]), while *SCMH1* was associated with stature phenotypes in European and African taurine breeds [40]. The fact that we identified a missense mutation with a relatively high F*_ST_* in the *SCMH1* gene and a signature of selection in this gene, unique for the KWH, suggests that this region is under ongoing selection in KWH and the Met392Val variant could be tested for marker-assisted selection in KWH. The other two genes found in KWH selection signatures and elevated F*_ST_* for missense SNP were *ARL11* (ADP ribosylation factor like GTPase 11) and *JDO2* (indoleamine 2,3-dioxygenase 2). In multiple studies, the *ARL11* was associated with body weight in chickens [41,42,43], suggesting that it could affect this phenotype in cattle. The top haplotype signature of selection specific to the KWH breed was observed in *THEGL* (spermatid protein-like), contributing to various stages of male gonad development, fertilisation, and embryonic development [44], suggesting that fertility phenotypes are under ongoing selection in the KWH. An elevated F*_ST_* supports this for a missense mutation in the *SLC9C1* (solute carrier family 9 member C1) gene observed in the point analysis, excluding Hereford and Kalmyk cattle samples. The *SLC9C1* is a sodium/proton exchanger associated with sperm mobility and spermatogenesis in cattle [45], humans, and mice and controls sperm motility through soluble adenylyl cyclase [46], suggesting that mutations in this gene with elevated F*_ST_* could be tested as markers for improving fertility traits in the KWH. The importance of this is supported by cases where the selection of economically important traits caused serious fertility issues in cattle breeds. One example is the selection for milk production in the American Holsten population, which led to fertility problems [47,48,49]. This indicates that future breeding programs should consider markers that could improve or maintain fertility, including those described above. The majority of the 42 missense variants identified in this study are good candidates to be tested in marker-assisted breeding strategies, as their genes show substantial enrichment in functional categories related to economically important traits.

An important limitation of our study is related to a relatively recent origin of the KWH breed, resulting in low differences in the frequency of individual alleles reflected in the point SNP analysis. A larger number of KWH samples from genetically different KWH populations could resolve this limitation to some extent, and this should be tried in the follow-up studies. By increasing the number of diverse samples, there is a chance that long haplotypes broken by recombination will be detected and additional point selection signatures become detectable. Because of the same limitation, we could expect that some of the 42 missense candidate variants are linked to those selected. The next endeavour we must focus on is the association analysis of the KWH phenotypes with these markers to confirm SNPs associated with commercial phenotypes.

## 5. Conclusions

In summary, our work has achieved this study’s goals, confirming the origins of KWH cattle from Hereford, Kalmyk, and native cattle. Our analysis shows that Hereford genetics, on average, had the highest contribution (45%) to the breed genomes. However, local cattle populations and hardy breeds could have impacted adaptive phenotypes, such as climatic adaptations and reproductive efficiency. Markers found in this study could help improve these traits if a follow-up association study confirms their effect/strong association with relevant phenotypes. Our results suggest that, in young composite breeds like the KWH, selection acting on haplotypes could hide the effects of selection acting on individual genetic variants. More generations need to pass or/and unrelated samples need to be used to break long ancestral haplotypes and see the effects of selection acting on individual mutations. This implies that genetic variants, even with elevated F*_ST_* found in this study, are strong candidates for use in marker-assisted selection and genetic predictions to improve the phenotypes of KWH. They should be tested in future efforts to enhance beef breeding programs in Kazakhstan.

## Figures and Tables

**Table 1:** Top candidate signatures of selection and genes identified by haplotype and window/point F*_ST_* analyses.

Signature of Selection	Method(s)	Breeds	Candidate Gene(s)	Missense Variant (F*_ST_* > 0.4)
6:67645166-68184415	hapFLK, F*_ST_*	KWH, HER *	*DCUN1D4 CWH43*	-
6:71907766-71969437	hapFLK, F*_ST_*	KWH	*THEGL*	-
6:68416511-68733563	hapFLK, F*_ST_*	KWH, HER	*SCFD2 RASL11B*	-
6:68754662-69049822	hapFLK, F*_ST_*	KWH, HER	*SCFD2*	-
12:19149453-19363604	hapFLK	KWH	*ARL11*	Val11Ala
6:83253647-83572456	hapFLK, F*_ST_*	KWH	*STAP1* *GNRHR CENPC*	-
3:104751571-105143542	hapFLK, F*_ST_*	KWH	*RNU6-419P* *SCMH1*	- Met392Val
10:54704096-54793283	hapFLK, F*_ST_*	KWH	*PRTG*	-
27:34758230-34908614	hapFLK, F*_ST_*	KWH	*ADAM18*	-
27:35061501-35168660	hapFLK, F*_ST_*	KWH	*IDO2*	Arg362Leu
6:66698083-66867714	hapFLK, F*_ST_*	KWH, HER	*TEC*	-
10:49394085-49429474	hapFLK, F*_ST_*	KWH, HER	*RORA*	-
6:66962751-67150552	hapFLK, F*_ST_*	KWH, HER	*SLAIN2*	-
17:313424-389767	hapFLK, F*_ST_*	KWH	*KLHL2*	-

* KWH—Kazakh Whiteheaded, HER—Hereford.

## Data Availability

The raw sequencing data for the Kazakh Whiteheaded animals are available from NCBI SRA under the BioProject accession numbers PRJNA1127033 and PRJNA762180.

## Supplementary Materials

The following supporting information can be downloaded at https://www.mdpi.com/article/10.3390/ani15030385/s1: Figure S1: Admixture analysis of 26 cattle breeds selected based on Hudson F*_ST_*; Table S1: Results of Hudson F*_ST_* analysis of KWH cattle and breeds from the 1000 Bull genomes project (≥10 samples + Kazakh cattle); Table S2: Samples used and excluded from the analysis; Table S3: HapFLK regions chosen on the basis of clustering and phylogenetic results with comparison to previous publications; Table S4: F*_ST_* analysis of KWH samples against Hereford, Kalmyk, and Altai samples; Table S5: F*_ST_* analysis of KWH samples against Kalmyk and Altai samples; Table S6: F*_ST_* analysis of KWH samples against Hereford and Kalmyk samples; Table S7: F*_ST_* analysis of KWH samples against Hereford and Altai samples; Table S8: Genes with missense mutations in Kazakh Whiteheaded cattle and F*_ST_* > 0.4 against Kalmyk and Altai samples; Table S9: Genes with missense mutations in Kazakh Whiteheaded cattle with F*_ST_* > 0.4 against Altai and Hereford samples; Table S10: DAVID enrichment analysis of genes with in hapFLK KWH regions; Table S11: DAVID enrichment analysis of genes in hapFLK in (KWH, HER) regions; Table S12: DAVID enrichment analysis of genes in hapFLK in (KWH, HER) and KWH regions; Table S13: DAVID enrichment analysis of missense variants with F*_ST_* > 0.4 when KWH was compared against Altai and Kalmyk samples; Table S14: Local ancestry inference for KWH genomes; Table S15: DAVID functional enrichment analysis in LAI gene set (Altai ancestry >0.5); Table S16: DAVID functional enrichment analysis in LAI gene set (Kalmyk ancestry >0.5); Table S17: DAVID functional enrichment analysis in LAI gene set (Hereford ancestry >0.6).
