# Supplementary material for: Resequencing Composite Kazakh Whiteheaded Cattle: Insights into Ancestral Breed Contributions, Selection Signatures, and Candidate Genetic Variants
Source: Animals (Basel). 2025 Jan 29;15(3):385. doi: 10.3390/ani15030385 (PMC11815988; doi:10.3390/ani15030385)
Supplement: Supplementary file 1 [file animals-15-00385-s001.zip › KWH_FigureS1.pptx]

## Slide 1
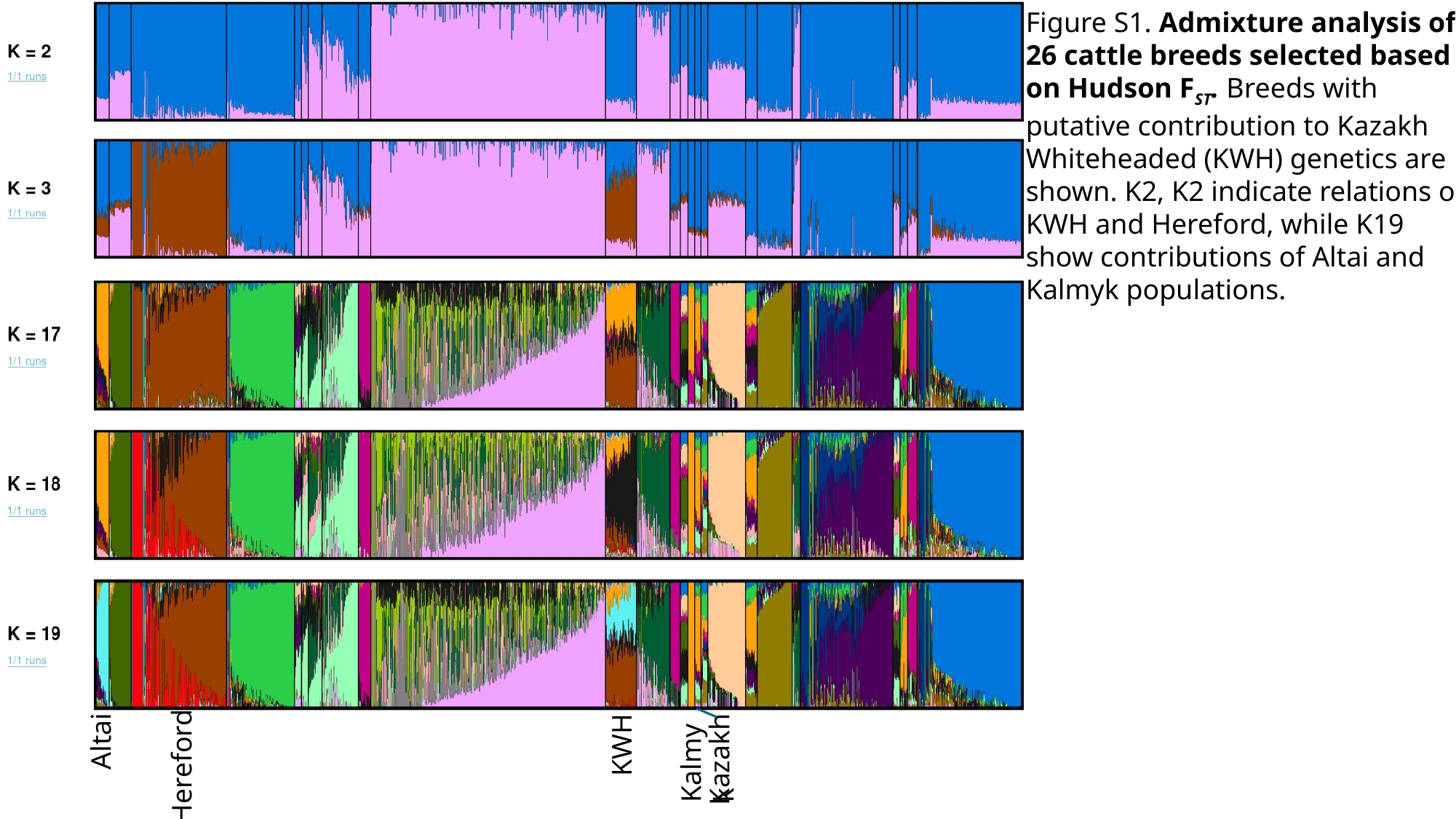

Figure S1. Admixture analysis of
26 cattle breeds selected based
on Hudson FST. Breeds with
putative contribution to Kazakh
Whiteheaded (KWH) genetics are
shown. K2, K2 indicate relations of
KWH and Hereford, while K19
show contributions of Altai and
Kalmyk populations.
Altai
KWH
Kalmyk
Kazakh
Hereford
